# Host-gut microbiota derived secondary metabolite mediated regulation of Wnt/β-catenin pathway: a potential therapeutic axis in IBD and CRC

**DOI:** 10.3389/fonc.2024.1392565

**Published:** 2024-04-19

**Authors:** Sushma S. Kumar, Ashna Fathima, Preeti Srihari, Trinath Jamma

**Affiliations:** Cell Signaling Laboratory, Department of Biological Sciences, Birla Institute of Technology & Science-Pilani Hyderabad Campus, Hyderabad, Telangana State, India

**Keywords:** gut microbiota, secondary metabolites, bile acids, inflammation, Wnt/β-catenin signaling, CRC

## Abstract

The intestinal tract encompasses one of the largest mucosal surfaces with a well-structured layer of intestinal epithelial cells supported by a network of underlying lamina propria immune cells maintaining barrier integrity. The commensal microflora in this environment is a major contributor to such functional outcomes due to its prominent role in the production of secondary metabolites. Of the several known metabolites of gut microbial origin, such as Short Chain Fatty Acids (SCFAs), amino acid derivatives, etc., secondary bile acids (BAs) are also shown to exhibit pleiotropic effects maintaining gut homeostasis in addition to their canonical role in dietary lipid digestion. However, dysbiosis in the intestine causes an imbalance in microbial diversity, resulting in alterations in the functionally effective concentration of these secondary metabolites, including BAs. This often leads to aberrant activation of the underlying lamina propria immune cells and associated signaling pathways, causing intestinal inflammation. Sustained activation of these signaling pathways drives unregulated cell proliferation and, when coupled with genotoxic stress, promotes tumorigenesis. Here, we aimed to discuss the role of secondary metabolites along with BAs in maintaining immune-gut homeostasis and regulation of inflammation-driven tumorigenesis with emphasis on the classical Wnt/β-Catenin signaling pathway in colon cancer.

## 1 Introduction

Inflammatory Bowel Diseases (IBD) are a collection of chronic inflammatory disorders associated with the gastrointestinal tract consisting of Crohn’s Disease (CD) and Ulcerative Colitis (UC) (1). The presence of IBD also increases the risk of development of colon cancer by 20% at later stages of life (2). Prolonged inflammation, along with other epigenetic factors and a dysregulated immune system, can contribute to the development of Colorectal Cancer (CRC) (2). According to WHO, CRC is the third most prevalent cancer worldwide after breast and lung cancer until 2020 (3). More than 50% of new cases of CRC were reported in Asia, followed by Europe and North America. It is predicted that if the situation persists, then the estimated number of cases will increase from 1.88 million in 2020 to 2.94 million in 2040 globally (4).

Current CRC treatment regimens include chemotherapy, T-cell boosting therapeutics, oncolytic viral treatments, and non-coding RNA therapy. However, these improved treatments did have long-term side effects that can affect quality of life. Up to 85% of survivors treated with Oxaliplatin develop some degree of sensory neuropathy (5). Another survey found that around 20% of patients undergoing chemotherapy experienced grade 3/4 severe toxicities. A smaller percentage (<1%) suffers fatal toxicity, resulting in severe diarrhea, neutropenia, thrombocytopenia, or cardiac symptoms (6). The increased resistance to non-coding RNA therapy over time also poses a major challenge (7). Therefore, despite the developments in biologics, surgery remains one of the major treatment strategies for CRC patients, implying the need for alternative therapies with minimal to no side effects.

## 2 The gut microbiome as a regulator of intestinal health: a quick overview

The intestinal microbial composition is closely associated with human health and disease. The human gut contains two compartments, the intestinal lumen and lamina propria, separated by the intestinal epithelial barrier. The luminal cavity is colonized by over 1000 species of microbes belonging to the domains *Archaea*, *Bacteria*, and *Eukarya*, which share a commensal relationship with cells of the host. The gut microbiome aids in various biological functions of the host system, such as fermentation of food, vitamin production, secondary metabolite synthesis, and regulation of immune responses (8). It has been reported that certain gut microbiota-derived secondary metabolites influence innate immune cells and non-hematopoietic components of the gut to maintain barrier integrity (9).

Prolonged disease conditions, a change in lifestyle and diet, and imprudent consumption of antibiotics result in gut microbial dysbiosis, subsequently disrupting intestinal homeostasis (9). The modern diet includes calorie-dense and nutritionally deficit options. Additionally, the increased consumption of ultra-processed foods (UPF) is also one of the leading factors contributing to the onset of IBD by reducing gut microbial diversity. Patients suffering from IBD, or gastrointestinal illness, along with medications, are often suggested a strict diet and healthy lifestyle. An appropriate dietary intervention can help in enhancing the effectiveness of the medication. Some dietary strategies have been found effective in improving disease activity and supporting clinical remission; however, some need further prospective evidence (10). For example, in a clinical study conducted in 2015, children suffering from Crohn’s disease were subject to a specific carbohydrate diet for 12-52 weeks. They showed reduced severity of the disease with respect to the Harvey-Bradshaw Index from 3.3 +/- 2.0 to 0.6 +/- 1.2 post-treatment (11).

In a healthy individual, the intestinal epithelial cell barrier can prevent the transmission of pathogens, proinflammatory substances, and antigens from the lumen to the internal environment (8). However, an imbalance in intestinal microbiota alters the tight intercellular junctions that allow pathogens and toxins (bacterial lipopolysaccharides, LPS) to cross the intestinal barrier, contributing to the activation of Pattern Recognition Receptors (PRRs) on lamina propria immune cells (12, 13). The intracellular signaling cascades triggered by these PRRs, which include Toll-like receptors (TLRs), RIG-I-like receptors (RLRs), NOD-like receptors (NLRs), and C-type lectin receptors (CLRs), upregulates the expression of inflammatory modulators. These modulators orchestrate the elimination of pathogens and affected cells. However, aberrant activation of this system also leads to the overproduction of immuno-oncogenic signals initiating tumorigenesis (13).

Cumulative studies illustrate that NLRs can negatively regulate cell differentiation and proliferation via the Wnt pathway in various cancers, including CRC (14). Similarly, TLR activation negatively regulates mesenchymal stem cell proliferation by disrupting canonical Wnt signaling by interrupting the expression of Wnt2, Wnt3, Wnt3a, and Wnt8 along with Frizzled Receptors (10). Wnt signaling is involved in the modulation of immune responses during inflammation, providing us with a potential drug target for CRC (12, 15). Therefore, through this review, we aim to shed light on the possible non-invasive methods to treat chronic intestinal inflammation and modulate the Wnt pathway using naturally occurring secondary metabolites of host gut-microbial origin.

## 3 Wnt signaling cascade and its role in intestinal cancer progression

CRC may result from one or more mechanisms such as chromosomal instability (CIN), CpG island methylator phenotype (CIMP), and microsatellite instability (MSI). The most studied mode of mechanistic progression is chromosomal instability, initiated by adenomatous polyposis coli (APC) mutations. Approximately 80% of CRC cases are a result of APC mutation. This mutation activates Wnt signaling mechanisms, increasing the transcription of several oncogenes (16) An interesting study conducted in 2020 revealed APC is also imperative for controlling Wnt-induced beta-catenin destruction complex recruitment in colonocytes to prevent aberrant cell proliferation and tumorigenesis (17), suggesting the involvement of Wnt signaling in CRC progression.

The Wnt/β-catenin pathway, Wnt/Ca2+ pathway, Wnt planar cell polarization pathway, and intracellular pathway that regulates spindle direction and asymmetric cell division are four major Wnt signaling pathways (18). The Wnt/β-catenin pathway displays a duality while modulating inflammation, possessing anti- and proinflammatory potential (14). In IBD’s pathophysiology, Wnt ligands secreted by activated immune cells bind to the Frizzled (Fzd), a G-protein coupled receptor, producing a proinflammatory tumor microenvironment (14, 19). Once Wnt ligands bind to membrane receptor Fzd and Lipoprotein-receptor related protein 5/6 (LRP5/6), they destabilize the β-Catenin degradation complex (GSK-3-β-APC-AXIN- β-Catenin). Accumulated β-Catenin translocates to the nucleus and triggers the TCF-4/LEF-1 (T cell factor/lymphoid enhancer factor) transcription factors to induce the expression of genes involved in cell cycle function and promote cell growth, differentiation, and metastasis (19).

A higher concentration of the Wnt ligands causes greater activation of the Wnt/β-Catenin signaling pathway, leading to increased cell proliferation. This uncontrolled cell proliferation or hypertrophy is followed by hyperplasia, causing an Epithelial-to-Mesenchymal (20) transition and increased cell motility, metastasis, and other related properties of cancer cells (21). Studies show that Wnt3a is the primary ligand involved in oral carcinogenesis, Wnt5a in breast tissue carcinogenesis, and Wnt3 is responsible for colon cancer proliferation (15, 21–23). Additionally, multiple types of cancer are known to be driven by uncontrolled expression of β-Catenin. β-Catenin expression is directly proportional to the depth of tumor infiltration (20). A swelling body of evidence suggests that β-Catenin inhibition suppresses tumor progression and recurrence.

During the clinical treatment of CRC, Wnt inhibitors are a common mode of therapy (24). The transcription factor SP1 (Specificity protein 1) is a crucial factor expressed in cell proliferation pathways (25). The direct interaction of SP1 with β-Catenin prevents the association of SP1 with degrading factors, thereby contributing to its stabilization (25). Interestingly, a study found suppression of the transcription factor SP1 by siRNAs truncated the growth of colon cancer stem cells (CCSCs) (21). Another transcription factor that promotes the proliferation of Wnt-driven colon cancer cells is SOX9. The regulation of gene expression by the Wnt/β-Catenin pathway results from the formation of a β-Catenin complex with the transcription factor TCF7 (T cell factor). TCF7 and SOX9 interact through nonDNA-contacting residues to produce a synergistic effect that encourages cancer cell proliferation (26). Inhibition of such factors can be a potential means of CRC therapeutics. These studies indicate that uncovering molecular targets within the Wnt/β-Catenin pathway will be capable of down-regulating CRC and related predisposing conditions such as IBD.

## 4 Gut microbiota derived secondary metabolites and their therapeutic potential in CRC

Culture-based studies show the dominance of *Bacteroidetes* and *Firmicutes* in the healthy gut, while *Actinobacteria*, *Proteobacteria*, and *Verrucomicrobia* are found in minor constituents. Reduction in diversity within the *Firmicutes* phylum is a major contributor to gut microbial dysbiosis causing IBD (8). Molecular cues of gut microbial origin regulating intestinal cell function are attributed to diversified small molecule metabolites (24). These metabolites are the intermediate or end products of host-gut bacterial metabolic processes. They are known to play a significant role in maintaining intestinal barrier integrity and intestinal immune homeostasis. Gut microbiota is widely involved in the metabolism of carbohydrates to generate SCFAs (27). Other majorly explored metabolites include tryptophan and indole derivatives, followed by primary and secondary BAs (27, 28).

Drastic imbalances in the composition of these metabolites have been observed in IBD and CRC patients. IBD patient fecal samples have a lower proportion of SCFA-producing bacteria, whereas mucolytic and pathogenic bacteria are found in abundance. Similarly, an increase in the population of sulfate-reducing bacteria, such as *Desulfovibrio*, is also found in IBD patient’s fecal samples. This increases the production of hydrogen sulfate, induces mucosal inflammation, and causes damage to the intestinal epithelial barrier (10). Moreover, IBD and IBD-associated cancers are known to cause malabsorption and reduction in the conversion of primary BAs to secondary BAs, thereby disrupting BA pool composition. Such changes pose a higher risk of infection as the mucosal integrity gets compromised (29).

Thus, their ability to behave as biomarkers and regulate metabolism and other homeostatic mechanisms makes them potential non-invasive therapeutic targets. (**Supplementary Table 1**).

### 4.1 Short chain fatty acids

Short Chain Fatty Acids (SCFAs) are crucial in maintaining intestinal barrier integrity, gut homeostasis, and colon health (30). These microbiota-derived SCFAs are the primary energy source for intestinal epithelial cells (IEC) in the digestive tract. The imbalance in SCFAs is known to contribute to intestinal inflammation and associated diseases (30). These SCFAs include butyrate, propionate, and acetate.

One of the significant SCFAs, butyrate, is produced by *Faecalibacterium prausnitzii*, *Clostridium leptum, and Eubacterium rectaleand*, among others, displays superior inhibitory efficacy against CRC proliferation (30). It is essential for human health as it is the primary energy source for colonocytes (31). Additionally, butyrate regulates CRC by inhibiting HDAC 1 and 3 in colon cancer cells and suppressing intestinal inflammation and ROS production (32). Butyrate activates GPR109A and inhibits Protein Kinase B and NF-κB signaling pathways to reverse intestinal epithelium barrier dysfunction (33). Furthermore, evidence shows that butyrate plays a vital role in controlling intestinal inflammation by stimulating the differentiation of Treg cells (34) and promoting an anti-tumor effect (35). It was also reported that *C. butyricum* species indirectly upregulates butyrate production, reduces the levels of β-Catenin, and regulates the Wnt pathway (36).

A study reported that butyrate facilitates M2 macrophage polarization. It was shown that ERK1/2 activation or blockade of Wnt secretion suppressed the beneficial effect of butyrate-primed macrophages on goblet cell function. Adoptive transfer of butyrate-induced M2 macrophages in a dextran sulfate sodium (DSS)-induced mice model of colitis showcased a significant improvement in mucosal layer integrity, mucus secretion, and goblet cell regeneration (37). It is also known that Butyrate stimulates bone formation via T Regulatory cell-mediated regulation of WNT10B expression (38).

A study by Beatrice et al. showed that butyrate inhibits CRC proliferation by autophagy-mediated degradation of β-Catenin. Apart from modulating cancer cell proliferation, the treatment with butyrate plays a significant role in autophagy. Interestingly, the study showed that butyrate promoted the binding between LC3 and β-Catenin, causing its sequestration. The ability of butyrate to inhibit the Wnt/β-Catenin pathway represents a new frontier of targeted cancer therapies (39).

**Figure 1:**
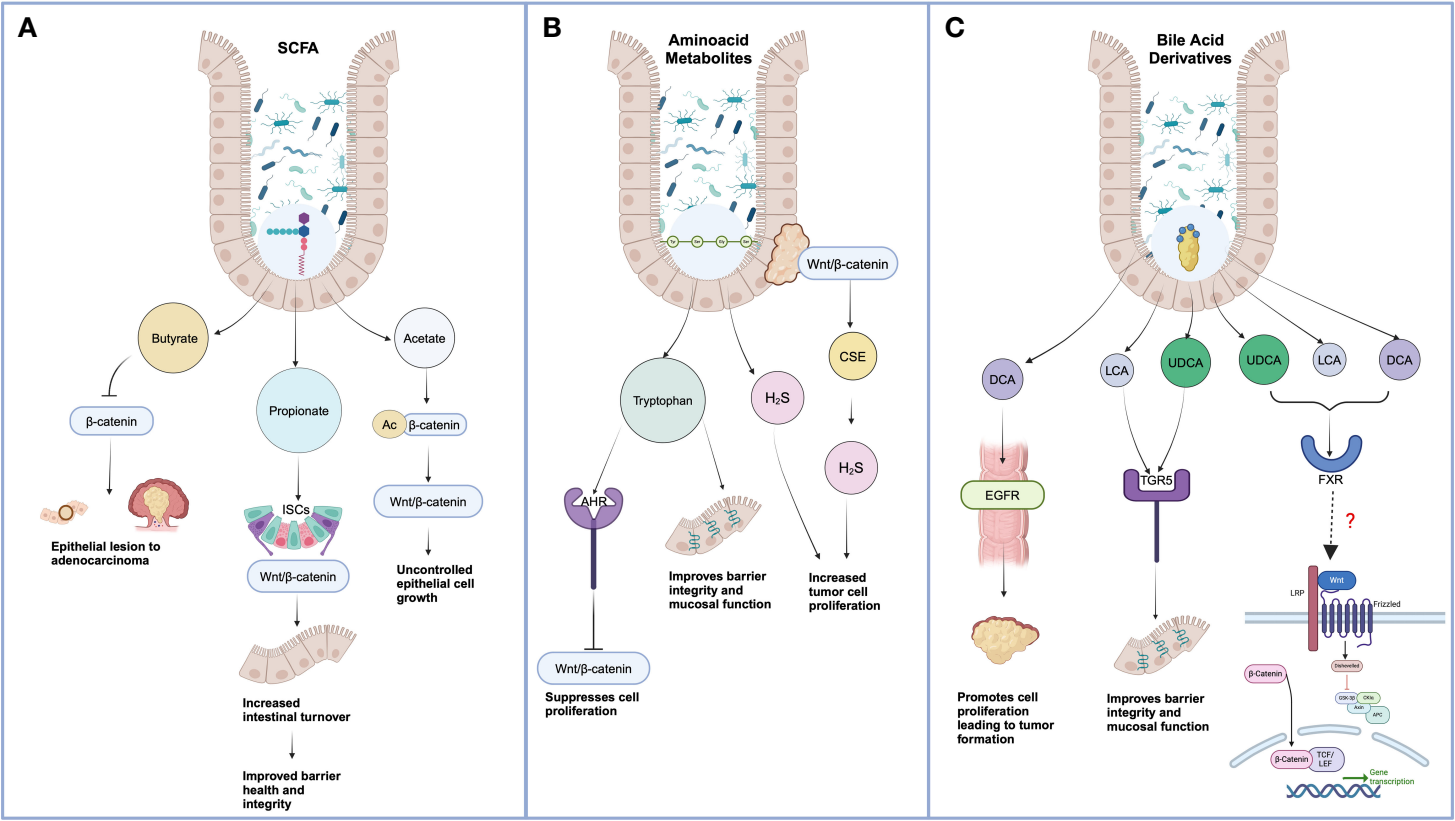
Effect of gut microbiota-derived metabolites on Wnt-mediated cancer progression. **(A)** Short-chain Chain Fatty Acids include Butyrate, Propionate, and Acetate. Excess butyrate treatment can promote proteasomal degradation of Wnt ligand β-Catenin mediated by autophagy marker LC3. When propionate synthesis reduces, cell differentiation and polarization diminish, leading to a lower cell turnover and increased aberrant intestinal cell growth. Acetate-mediated acetylation of β-Catenin facilitates a decrease of SOX-1, allowing unchecked Wnt-mediated cell proliferation. **(B)** Lower tryptophan metabolism impairs the integrity of tight junctions and regulates Wnt metabolism by targeting Wnt 3 and β-Catenin. H2S nourishes aberrant cell proliferation via the Wnt pathway. **(C)** Bile Acids bind to the FXR receptor, resulting in the inhibition of the Wnt/β-Catenin pathway. However, few direct co-relations exist between BAs and Wnt signaling ligands and receptors. *Created in BioRender.com).

Similarly, propionate, produced by *Veillonella parvula, Bacteroides eggerthii, and Bacteroides fragilis* in the gut, regulates intestinal homeostasis by promoting turnover of the epithelial cells and promoting barrier integrity (40). As a result, stem cells in intestinal crypts differentiate through the Wnt signaling pathway to replenish lost cells. The absence of propionate results in intestinal disbalance, triggering the unregulated proliferation of IECs (30). Valproic acid (VPA) was able to stimulate the differentiation of neuronal stem cells by activating Wnt3a and β-Catenin (18, 41). The fatty acid acetate aids in the acetylation of β-catenin, reducing the Wnt inhibitor SOX-1 and potentially increasing cell proliferation (14). Recently, another study showed β-hydroxybutyrate is capable of suppressing cancer, by inhibiting EMT via the Wnt/β-Catenin pathway (42). (**Figure 1A**).

### 4.2 Amino acid metabolites

Numerous amino acid metabolites, including hydrogen sulfide (H_2_S) and indole metabolites, are produced due to the fermentation of proteins by the gut microbiota (28). Several studies have shown that tryptophan (Trp), mainly produced by *E. coli*, can downregulate cell proliferation by suppressing the Wnt signaling pathway, implying targeting tryptophan metabolism is a method of CRC treatment (42, 43). A clinical study was conducted on 117 participants comprising 79 CRC patients and 38 age- sex-and body mass index (BMI) matched healthy controls. It was observed that the indole/tryptophan ratio in fecal matter positively correlated to the mRNA expression of tight junction proteins like Zona Occuladins-1 in colon tissue samples collected from the respective participants, suggesting the involvement of Trp metabolites in the tumorigenesis of CRC in humans (43). Several studies have highlighted the role of wnt signaling in shaping immune cell functions. One of the key mechanisms by which Wnt-β-catenin signaling in DCs promotes immune suppression is through the induction of an immunoregulatory enzyme, IDO, thereby causing the degradation of the essential amino acid tryptophan into kynurenines (44). A study found that 1-Methyl-D-tryptophan Reduces Tumor CD133^+^ cells, Wnt/β-catenin and NF-κβp65 in Murine Pancreatic Adenocarcinoma.1-Methyl-D-tryptophan significantly modulates the regulatory cytokines in the tumor microenvironment, which significantly inhibited tumor growth and tumor immune escaping potency (45).

Studies indicate another predominant amino acid metabolite, H_2_S, an energy source for the metabolism of the colonic epithelium (46). H_2_S is produced by the action of *Desulfovibrio*, *Escherichia*, *Bilophila*, *Porhyromonas*, *Prevotella*, *Corynebacterium*, *Veillonella*, *Helicobacter*, and *Clostridium* on amino acids (27). Cell culture studies in HT 29 cells discovered the cytotoxic and genotoxic effects of H_2_S produced by sulfate-reducing bacteria. However, there has been conflicting data on the inhibitory and stimulatory effects of H_2_S on the proliferation and inflammation of CRC cells (47). Upon further analysis in Human Colon Cancer cell line SW480, the study found that the Wnt/β-Catenin pathway regulates Cystathionine-γ-lyase (CSE) on a transcriptional level, upon secretion responsible for increasing H_2_S liberation. Furthermore, when tumors were xenografted into nude mice models with a CSE/H_2_S knockdown, their tumor growth was reduced, implying that H_2_S plays a role in increasing colon cancer (48) (**Figure 1B**).

### 4.3 Bile acids

Another well-known host-gut microbiota-derived metabolite of interest are secondary BAs with numerous unknown functions other than role in dietary lipid digestion. BAs are the end-product of cholesterol metabolism generated in the liver by a chain of enzymatic reactions organized in two main metabolic pathways, known as “classic” and “alternative” (49). These liver pathways generate mainly two primary BAs, i.e., cholic acid and chenodeoxycholic acid (CA and CDCA). In hepatocytes, these primary BAs are conjugated with glycine (G) or taurine (T), giving rise to the bile salts. Conjugated BAs are secreted in the intestine, becoming the substrate of an array of bacterial enzymes (49). 7α-dehydroxylation of the OH in the C7 position, a reaction mediated by 7α-hydroxylase expressing bacteria such as *Clostridium* and *Eubacterium*, gives rise to two secondary BAs, i.e., mono-hydroxylated BAs like LCA from CDCA, and 3α-12α-di-hydroxylated BAs like DCA from CA. Additionally, the C7 β-epimerization of CDCA by *Bacteroides, Clostridium, Escherichia, Eubacterium*, and others originates the 7β epimer of CDCA, i.e., the 3α,7β-dihydroxy-5β-cholanoic acid, known as ursodeoxycholic acid (UDCA) (47, 48). The large majority of BA species that reach the terminal ileum are reabsorbed by the intestinal epithelial cells (IEC) and transported back to the liver through the portal vein, completing a cycle in the so-called “entero-hepatic circulation” (49).

#### 4.3.1 Therapeutic potential of BAs

BAs regulate mucosal homeostasis and inflammation by interacting directly with a family of receptors known as bile acid-activated receptors or bile acid receptors (BAR), which include Takeda G protein-coupled receptor 5 (TGR5) and nuclear receptors that include the Farnesoid X Receptor (FXR) and Vitamin D Receptor (VDR) (50). BA signaling is known to suppress the proinflammatory phenotype of intestinal cells by the reduced release of TNF-α, IL-1β, IL-6, or IL-12. Studies have also reported that BA stimulates the production of anti-inflammatory cytokines, promoting epithelial barrier renewal (28).

A study reported that secondary BAs, such as LCA’s derivatives, regulate the differentiation of Treg cells, contributing to the suppression of inflammation, maintaining immune homeostasis, and hence, predisposing stages of cancers like CRC (51). LCA is reported to activate VDR on CaCo-2 cells and significantly reduce IL-1β -induced IL-8 secretion by blocking NF-κB inflammatory signaling (52). Kubota et al., in their studies, found VDR mediated the attenuation of Dextran Sulfate Sodium (DSS) induced Colitis in mice fed with LCA (53). Oral administration of LCA suppressed histological injury in an early phase of DSS-induced Colitis in Vdr+/- mice, whereas no significant impact was observed on Vdr-/- mice, suggesting the physiological role of the LCA–VDR axis in intestinal homeostasis (53). Additionally, LCA-dependent PXR activation in epithelial cells promotes TGFβ expression and reduces TLR4-dependent proinflammatory cytokines production by diminishing TLR4 mRNA stability (54). TGR5 is one of the receptors activated by multiple BAs, with LCA being its most potent natural agonist (55). A study found that LCA-induced activation of TGR5 reduces adaptive immune response as there is increased recruitment of NK cells. Another study found that LCA stimulated intestinal epithelial growth in an organoid, as indicated by the increased expression of an intestinal stem cell marker. However, this improved barrier regeneration was lost when LCA was administered to a *Tgr5-/-* organoid, indicating that LCA-associated TGR5 activation is crucial for barrier integrity (55).

Multiple studies have reported the therapeutic role of another secondary BA, UDCA, in Colitis and colitis-associated cancer. UDCA exerts anti-inflammatory and cytoprotective effects in the AOM-DSS-induced colitis mouse model (55). UDCA has also been shown to prevent colon inflammation in rats treated with 2,4,6-trinitrobenzene sulfonic acid (56). Interestingly, deficiency or absence of the TGR5 receptor significantly reduces the modulatory effect of UDCA, both *in vitro* and *in vivo*. He et al. and other studies highlight that UDCA treatment can contribute to intestinal homeostasis by enhancing the intestinal mucosal layer, maintaining epithelial cell integrity, modulating the gut microenvironment, and attenuating intestinal inflammation (55). The collective observation suggests that elucidating the relationship between UDCA and the gut microbiome can be a novel therapeutic strategy for inflammation and inflammation-driven cancer.

DCA has been known to play a significant role in the induction of CRC development. *In vivo* experiments with APC^min/+^ mice suggested that DCA contributes to CRC tumorigenesis by activating EGFR to promote a hyperproliferative effect on colorectal mucosa in DCA-fed mice (57). Ji-Yao et al. reported that oral administration of DCA to germ-free mice increased colonic Rspo3 mRNA levels, which function as ligands for LGR4 and LGR5 and potentiate the activation of the Wnt pathway. In primary myofibroblasts, DCA increases Rspo3 mRNA via TGR5 and mediates high-fat diet-induced intestinal epithelial proliferation (58). However, the impact of therapeutic BAs like UDCA and LCA and its derivatives on wnt regulation are largely unexplored. (**Figure 1C**).

#### 4.3.2 The cross-talk between Wnt/β-catenin and bile acids

Recent evidence indicates that the Wnt/β-catenin pathway regulates bile homeostasis, including bile synthesis, modification, and transport. Cholesterol synthesis occurs predominantly in periportal hepatocytes (59). CYP7A1 and CYP27, crucial rate-limiting enzymes of BA synthesis, are localized in the perivenous zone of the liver lobule and coincident with β-catenin activation. The close relationship between the two processes was seen in β-catenin KO mice subjected to a methionine and choline-deficient diet, identified by macro vesicular steatosis and fibrosis. Liver-specific β-catenin deletion resulted in increased steatosis, higher hepatic cholesterol accumulation, and jaundice, likely due to defects in cholesterol to bile conversion mechanism and the bile export system. Additionally, conditional β-catenin KO had higher hepatic total BA levels on methionine and choline-deficient and control diets, indicative of basal abnormalities in bile metabolism without β-catenin (60).

Chromatin immunoprecipitation (ChIP) assays showed that CYP27 is a transcriptional target of β-catenin. Similarly, β-catenin KO and LRP5/6 KO models had significantly suppressed expression of CYP7A1, suggesting the involvement of β-catenin in BA metabolism. Interestingly, further studies have found that the β-catenin interacts with FXR, a nuclear receptor that regulates the expression of CYP7A1 and BA efflux transporters. FXR deficiency increases epithelial permeability to luminal bacteria, thereby promoting Wnt/β-catenin signaling, and increasing intestinal inflammation (61).

The crosstalk between Wnt/β-catenin ligands and members of the nuclear receptor (NR) family has been considered a clinically and developmentally important research area of cancer biology. Mao J. et al., in their study, demonstrated that FXR knockdown promotes β-catenin/TCF4 complex formation and, subsequently, its binding ability to the corresponding promoter. Their data indicates a novel mechanism through which FXR expression is mediated during tumor progression, involving the Wnt pathway. Additionally, hepatic bile acid synthesis is downregulated by the activation of the FXR-FGF15/19 signaling pathway (62). Thus, FXR represents a novel Wnt signaling pathway modulator and a potential Wnt signaling cascade molecular target that may be exploited to achieve anti-tumor effects (63).

## 5 Conclusion/discussion

The role of Wnt signaling in tumorigenesis is predominantly studied in colorectal cancer, where several studies suggest targeting Wnt/β catenin to regulate tumor progression. However, a therapeutic treatment targeting the canonical Wnt pathway achieving efficacy and safety remains a major challenge. Considering the role of FXR in Wnt regulation and the ability of some BAs to activate FXR, understanding the downstream mechanism opens doors to promising hypotheses exploring the impact of BAs via the BAR in regulating pathogenic Wnt signaling and immune modulation in the intestinal inflammation and associated cancers. As new studies describing such processes and our understanding of signaling mechanisms deepen, we must screen for direct interactions between BAs and Wnt pathways with the goal of maintaining intestinal homeostasis. Overall, the development of novel combinatorial therapeutics of natural origin capable of reducing the risk of side effects and improving the treatment outcome in CRC and predisposing IBD is an essential stride.

## Author contributions

SSK: Writing – review & editing, Writing – original draft, Conceptualization. AF: Writing – review & editing, Writing – original draft, Conceptualization. PS: Writing – review & editing. TJ: Writing – review & editing, Supervision, Funding acquisition, Conceptualization.
