# Supplementary material for: Host-gut microbiota derived secondary metabolite mediated regulation of Wnt/β-catenin pathway: a potential therapeutic axis in IBD and CRC
Source: Front Oncol. 2024 Apr 19;14:1392565. doi: 10.3389/fonc.2024.1392565 (PMC11066261; doi:10.3389/fonc.2024.1392565)
Supplement: Supplementary file 1 [file Table_1.docx]

**Host-Gut Microbiota Derived Secondary Metabolite mediated regulation of Wnt/β-catenin pathway: A Potential Therapeutic Axis in IBD and CRC.**

**Sushma S Kumar^#1^, Ashna Fathima^#1^, Preeti Srihari^1^, Trinath Jamma^1*^**

^1^ Cell Signaling Laboratory, Department of Biological Sciences, Birla Institute of Technology & Science-Pilani Hyderabad Campus, Jawahar Nagar, Shameerpet Mandal, Hyderabad, Telangana State 500078, India

***Corresponding author:** *trinath@hyderabad.bits-pilani.ac.in;* [*karunya.friend@gmail.com*](mailto:karunya.friend@gmail.com)*;*

# Equal contribution

**Supplementary Table 1:**

| Metabolites | | Gut microbiota | References |
| --- | --- | --- | --- |
| SCFA | Butyrate | Bacteroides, Staphylococcus, Coprococcus,  Clostridium, Roseburia, Faecalibacterium,  Fusobacteria, Collinsella, Eubacterium,  Enterococcus, Prevotella, Escherichia | Feng W et al., 2018(1)  Cheng et al., 2022(2)  Meyers et al., 2022(3)  Wu Y et al., 2021(4)  Panattoni et al., 2022(5)  Deleu et al., 2021(6)  Rudin D et al., 2021(7)  Gomez Arango et al., 2018(8)  Anita Slavica et al., 2015(9)  Ana Nogal et al., 2021(10)  Yaxin Chen et al., 2022(11)  Betancur-Murillo et al., 2023(12) |
|  | Propionate | Bacteroides, Campylobacter, Staphylococcus,  Coprococcus, Roseburia, Enterococcus,  Ruminococcus. Akkermansia, Prevotella |  |
|  | Acetate | Bacteroides, Campylobacter, Staphylococcus,  Bifidobacterium, Lactobacillus, Enterococcus,  Fusobacteria, Ruminococcus, Collinsella,  Escherichia, Akkermansia, Prevotella |  |
| Secondary BAs | DCA | Bacteroides, Bifidobacterium, Clostridium,  Roseburia, Faecalibacterium, Collinsella,  Prevotella | Zhou C et al., 2023(13)  Baorong Jiang et al., 2022(14)  Min Yang et al., 2021(15)  Bossche et al., 2017(16)  Lucas et al., 2021(17)  Li X et al., 2022(18)  Bustamante et al., 2022(19)  Xiaohua Guo et al., 2022(20)  Taylor SA et al., 2018(21)  Zhao C et al., 2023(22)  Jie, Zhuye et al., 2023(23) |
|  | LCA | Bacteroides, Coprococcus, Clostridium,  Roseburia, Faecalibacterium, Enterococcus,  Fusobacteria, Collinsella, Eubacterium,  Escherichia, Prevotella |  |
|  | UDCA | Bacteroides, Clostridium, Faecalibacterium,  Lactobacillus, Fusobacteria, Ruminococcus,  Collinsella, Eubacterium, Escherichia,  Akkermansia |  |
| Amino acid derivatives | Indole | Bacteroides, Bifidobacterium, Coprococcus,  Clostridium, Roseburia, Lactobacillus,  Enterococcus, Fusobacteria, Ruminococcus,  Collinsella, Eubacterium, Escherichia,  Prevotella | Xuewei Ye et al., 2022(24)  Harrisham Kaur et al., 2019(25)  Jin-Hyung Lee et al., 2010(26)  Fleur Notting et al., 2023(27)  Marta Wlodarska et al., 2017(28)  Charles Darkoh et al., 2019(29)  Kari Erik Murros, 2022(30)  Gang Li et al., 2013(31)  Takuma Sakurai et al., 2019(32)  Ratika Sehgul et al., 2022(33)  Takako Sasaki-Imamura et al., 2010(34)  Kai Nie et al., 2021(35)  Theresa L. Montgomery et al., 2022(36)  Maria J. Villanueva-Millan et al., 2022 (37)  Mireia Lopez-Siles et al., 2017 (38) |
|  | Tryptophan | Bacteroides, Coprococcus, Faecalibacterium  Enterococcus, Fusobacteria, Collinsella  Escherichia, Akkermansia |  |
|  | H2S | Bacteroides, Staphylococcus, Clostridium  Fusobacteria, Ruminococcus, Collinsella  Escherichia, Akkermansia, Prevotella |  |

References:

1. Feng, W., Ao, H., & Peng, C. (2018, November 23). *Gut Microbiota, Short-Chain Fatty Acids, and Herbal Medicines*. Frontiers in Pharmacology. https://doi.org/10.3389/fphar.2018.01354
2. Cheng, Y., Liu, J., & Ling, Z. (2022). Short-chain fatty acids-producing probiotics: A novel source of psychobiotics. *Critical reviews in food science and nutrition*, *62*(28), 7929–7959. <https://doi.org/10.1080/10408398.2021.1920884>
3. Meyers, G. R., Samouda, H., & Bohn, T. (2022, December 16). *Short Chain Fatty Acid Metabolism in Relation to Gut Microbiota and Genetic Variability*. Nutrients. https://doi.org/10.3390/nu14245361
4. Wu, Y., Xu, H., Tu, X., & Gao, Z. (2021, September 28). *The Role of Short-Chain Fatty Acids of Gut Microbiota Origin in Hypertension*. Frontiers in Microbiology. https://doi.org/10.3389/fmicb.2021.730809
5. Panattoni, A., Calvigioni, M., Benvenuti, L., D’Antongiovanni, V., Pellegrini, C., Di Salvo, C., Mazzantini, D., Celandroni, F., Fornai, M., Antonioli, L., & Ghelardi, E. (2022, December 16). *The administration of Enterococcus faecium SF68 counteracts compositional shifts in the gut microbiota of diet-induced obese mice*. Frontiers in Microbiology. https://doi.org/10.3389/fmicb.2022.1054097
6. Deleu, S., Machiels, K., Raes, J., Verbeke, K., & Vermeire, S. (2021). Short chain fatty acids and its producing organisms: An overlooked therapy for IBD?. *EBioMedicine*, *66*, 103293. <https://doi.org/10.1016/j.ebiom.2021.103293>
7. Dahlstrand Rudin, A., Khamzeh, A., Venkatakrishnan, V., Basic, A., Christenson, K., & Bylund, J. (2021). Short chain fatty acids released by Fusobacterium nucleatum are neutrophil chemoattractants acting via free fatty acid receptor 2 (FFAR2). *Cellular microbiology*, *23*(8), e13348. <https://doi.org/10.1111/cmi.13348>
8. Gomez-Arango, L. F., Barrett, H. L., Wilkinson, S. A., Callaway, L. K., McIntyre, H. D., Morrison, M., & Dekker Nitert, M. (2018). Low dietary fiber intake increases Collinsella abundance in the gut microbiota of overweight and obese pregnant women. *Gut microbes*, *9*(3), 189–201. <https://doi.org/10.1080/19490976.2017.1406584>
9. Slavica, A., Trontel, A., Jelovac, N., Kosovec, Ž., Šantek, B., & Novak, S. (2015). Production of lactate and acetate by Lactobacillus coryniformis subsp. torquens DSM 20004(T) in comparison with Lactobacillus amylovorus DSM 20531(T). *Journal of biotechnology*, *202*, 50–59. <https://doi.org/10.1016/j.jbiotec.2015.01.014>
10. Nogal, A., Valdes, A. M., & Menni, C. (2021). The role of short-chain fatty acids in the interplay between gut microbiota and diet in cardio-metabolic health. *Gut microbes*, *13*(1), 1–24. <https://doi.org/10.1080/19490976.2021.1897212>
11. Chen, Y., Liu, Y., Wang, Y., Chen, X., Wang, C., Chen, X., Yuan, X., Liu, L., Yang, J., & Zhou, X. (2022). Prevotellaceae produces butyrate to alleviate PD-1/PD-L1 inhibitor-related cardiotoxicity via PPARα-CYP4X1 axis in colonic macrophages. *Journal of experimental & clinical cancer research : CR*, *41*(1), 1. <https://doi.org/10.1186/s13046-021-02201-4>
12. Betancur-Murillo, C. L., Aguilar-Marín, S. B., & Jovel, J. (2022). Prevotella: A Key Player in Ruminal Metabolism. *Microorganisms*, *11*(1), 1. <https://doi.org/10.3390/microorganisms11010001>
13. Zhou, C., Wang, Y., Li, C., Xie, Z., & Dai, L. (2023, April 13). *Amelioration of Colitis by a Gut Bacterial Consortium Producing Anti-Inflammatory Secondary Bile Acids*. Microbiology Spectrum. https://doi.org/10.1128/spectrum.03330-22
14. Jiang, B., Yuan, G., Wu, J., Wu, Q., Li, L., & Jiang, P. (2022, March 1). *Prevotella copri ameliorates cholestasis and liver fibrosis in primary sclerosing cholangitis by enhancing the FXR signalling pathway*. Biochimica Et Biophysica Acta (BBA) - Molecular Basis of Disease. https://doi.org/10.1016/j.bbadis.2021.166320
15. Yang, M., Gu, Y., Li, L., Li, T., Song, X., Sun, Y., Cao, X., Wang, B., Jiang, K., & Cao, H. (2021, September 9). *Bile Acid–Gut Microbiota Axis in Inflammatory Bowel Disease: From Bench to Bedside*. Nutrients. https://doi.org/10.3390/nu13093143
16. Van den Bossche, L., Hindryckx, P., Devisscher, L., Devriese, S., Van Welden, S., Holvoet, T., Vilchez-Vargas, R., Vital, M., Pieper, D. H., Vanden Bussche, J., Vanhaecke, L., Van de Wiele, T., De Vos, M., & Laukens, D. (2017). Ursodeoxycholic Acid and Its Taurine- or Glycine-Conjugated Species Reduce Colitogenic Dysbiosis and Equally Suppress Experimental Colitis in Mice. *Applied and environmental microbiology*, *83*(7), e02766-16. <https://doi.org/10.1128/AEM.02766-16>
17. Lucas, L. N., Barrett, K., Kerby, R. L., Zhang, Q., Cattaneo, L. E., Stevenson, D., Rey, F. E., & Amador-Noguez, D. (2021). Dominant Bacterial Phyla from the Human Gut Show Widespread Ability To Transform and Conjugate Bile Acids. *mSystems*, e0080521. Advance online publication. <https://doi.org/10.1128/mSystems.00805-21>
18. Li, X., Wang, X., Wang, Z., Zhang, M., Wang, S., Xiang, Z., Pan, H., & Li, M. (2022, May 11). *The Relationship Between Gut Microbiome and Bile Acids in Primates With Diverse Diets*. Frontiers in Microbiology. https://doi.org/10.3389/fmicb.2022.899102
19. Bustamante, J. M., Dawson, T., Loeffler, C., Marfori, Z., Marchesi, J. R., Mullish, B. H., Thompson, C. C., Crandall, K. A., Rahnavard, A., Allegretti, J. R., & Cummings, B. P. (2022, December 7). *Impact of Fecal Microbiota Transplantation on Gut Bacterial Bile Acid Metabolism in Humans*. Nutrients. https://doi.org/10.3390/nu14245200
20. Guo, X., Okpara, E. S., Hu, W., Yan, C., Wang, Y., Liang, Q., Chiang, J. Y., & Han, S. (2022, July 28). *Interactive Relationships between Intestinal Flora and Bile Acids*. International Journal of Molecular Sciences. https://doi.org/10.3390/ijms23158343
21. Taylor, S. A., & Green, R. M. (2018). Bile Acids, Microbiota, and Metabolism. *Hepatology (Baltimore, Md.)*, *68*(4), 1229–1231. <https://doi.org/10.1002/hep.30078>
22. Zhao, C., Wu, K., Hao, H., Zhao, Y., Bao, L., Qiu, M., He, Y., He, Z., Zhang, N., Hu, X., & Fu, Y. (2023). Gut microbiota-mediated secondary bile acid alleviates Staphylococcus aureus-induced mastitis through the TGR5-cAMP-PKA-NF-κB/NLRP3 pathways in mice. *NPJ biofilms and microbiomes*, *9*(1), 8. <https://doi.org/10.1038/s41522-023-00374-8>
23. Jie, Z., Zhu, Q., Zou, Y., Wu, Q., Qin, M., He, D., Lin, X., Tong, X., Zhang, J., Jie, Z., Luo, W., Xiao, X., Chen, S., Wu, Y., Guo, G., Zheng, S., Li, Y., Lai, W., Yang, H., Wang, J., … Zhong, S. (2023). A consortium of three-bacteria isolated from human feces inhibits formation of atherosclerotic deposits and lowers lipid levels in a mouse model. *iScience*, *26*(6), 106960. <https://doi.org/10.1016/j.isci.2023.106960>
24. Ye, X., Li, H., Anjum, K., Zhong, X., Miao, S., Zheng, G., Liu, W., & Li, L. (2022). Dual Role of Indoles Derived From Intestinal Microbiota on Human Health. *Frontiers in immunology*, *13*, 903526. <https://doi.org/10.3389/fimmu.2022.903526>
25. Kaur, H., Bose, C., & Mande, S. S. (2019, December 18). *Tryptophan Metabolism by Gut Microbiome and Gut-Brain-Axis: An in silico Analysis*. Frontiers in Neuroscience. https://doi.org/10.3389/fnins.2019.01365
26. Lee, J. H. (2010, July 1). *Indole as an intercellular signal in microbial communities*. Fems Microbiology Reviews. https://doi.org/10.1111/j.1574-6976.2009.00204.x
27. Notting, F., Pirovano, W., Sybesma, W., & Kort, R. (2023, January 1). *The butyrate-producing and spore-forming bacterial genus Coprococcus as a potential biomarker for neurological disorders*. Gut Microbiome. https://doi.org/10.1017/gmb.2023.14
28. Wlodarska, M., Luo, C., Kolde, R., d'Hennezel, E., Annand, J. W., Heim, C. E., Krastel, P., Schmitt, E. K., Omar, A. S., Creasey, E. A., Garner, A. L., Mohammadi, S., O'Connell, D. J., Abubucker, S., Arthur, T. D., Franzosa, E. A., Huttenhower, C., Murphy, L. O., Haiser, H. J., Vlamakis, H., … Xavier, R. J. (2017). Indoleacrylic Acid Produced by Commensal Peptostreptococcus Species Suppresses Inflammation. *Cell host & microbe*, *22*(1), 25–37.e6. <https://doi.org/10.1016/j.chom.2017.06.007>
29. Darkoh, C., Plants-Paris, K., Bishoff, D., & DuPont, H. L. (2019). Clostridium difficile Modulates the Gut Microbiota by Inducing the Production of Indole, an Interkingdom Signaling and Antimicrobial Molecule. *mSystems*, *4*(2), e00346-18. <https://doi.org/10.1128/mSystems.00346-18>
30. Murros K. E. (2022). Hydrogen Sulfide Produced by Gut Bacteria May Induce Parkinson's Disease. *Cells*, *11*(6), 978. <https://doi.org/10.3390/cells11060978>
31. Li, G., & Young, K. D. (2013). Indole production by the tryptophanase TnaA in Escherichia coli is determined by the amount of exogenous tryptophan. *Microbiology (Reading, England)*, *159*(Pt 2), 402–410. <https://doi.org/10.1099/mic.0.064139-0>
32. Sakurai, T., Odamaki, T., & Xiao, J. Z. (2019). Production of Indole-3-Lactic Acid by *Bifidobacterium* Strains Isolated fromHuman Infants. *Microorganisms*, *7*(9), 340. <https://doi.org/10.3390/microorganisms7090340>
33. Sehgal, R., de Mello, V. D., Männistö, V., Lindström, J., Tuomilehto, J., Pihlajamäki, J., & Uusitupa, M. (2022). Indolepropionic Acid, a Gut Bacteria-Produced Tryptophan Metabolite and the Risk of Type 2 Diabetes and Non-Alcoholic Fatty Liver Disease. *Nutrients*, *14*(21), 4695. <https://doi.org/10.3390/nu14214695>
34. Sasaki-Imamura, T., Yano, A., & Yoshida, Y. (2010). Production of indole from L-tryptophan and effects of these compounds on biofilm formation by Fusobacterium nucleatum ATCC 25586. *Applied and environmental microbiology*, *76*(13), 4260–4268. <https://doi.org/10.1128/AEM.00166-10>
35. Nie, K., Ma, K., Luo, W., Shen, Z., Yang, Z., Xiao, M., Tong, T., Yang, Y., & Wang, X. (2021). *Roseburia intestinalis*: A Beneficial Gut Organism From the Discoveries in Genus and Species. *Frontiers in cellular and infection microbiology*, *11*, 757718. <https://doi.org/10.3389/fcimb.2021.757718>
36. Montgomery, T. L., Eckstrom, K., Lile, K. H., Caldwell, S., Heney, E. R., Lahue, K. G., D’Alessandro, A., Wargo, M. J., & Krementsov, D. N. (2022, November 23). *Lactobacillus reuteri tryptophan metabolism promotes host susceptibility to CNS autoimmunity*. Microbiome. https://doi.org/10.1186/s40168-022-01408-7
37. Villanueva-Millan, M. J., Leite, G., Wang, J., Morales, W., Parodi, G., Pimentel, M. L., Barlow, G. M., Mathur, R., Rezaie, A., Sanchez, M., Ayyad, S., Cohrs, D., Chang, C., Rashid, M., Hosseini, A., Fiorentino, A., Weitsman, S., Chuang, B., Chang, B., Pichetshote, N., … Pimentel, M. (2022). Methanogens and Hydrogen Sulfide Producing Bacteria Guide Distinct Gut Microbe Profiles and Irritable Bowel Syndrome Subtypes. *The American journal of gastroenterology*, *117*(12), 2055–2066. <https://doi.org/10.14309/ajg.0000000000001997>
38. López-Siles, M., Duncan, S. H., Garcia‐Gil, L. J., & Martínez-Medina, M. (2017, January 3). *Faecalibacterium prausnitzii: from microbiology to diagnostics and prognostics*. The ISME Journal. https://doi.org/10.1038/ismej.2016.176
